# Subcortical Intermittent Theta-Burst Stimulation (iTBS) Increases Theta-Power in Dorsolateral Prefrontal Cortex (DLPFC)

**DOI:** 10.3389/fnins.2020.00041

**Published:** 2020-01-31

**Authors:** J. Nicole Bentley, Zachary T. Irwin, Sarah D. Black, Megan L. Roach, Ryan J. Vaden, Christopher L. Gonzalez, Anas U. Khan, Galal A. El-Sayed, Robert T. Knight, Barton L. Guthrie, Harrison C. Walker

**Affiliations:** ^1^Department of Neurosurgery, University of Alabama at Birmingham, Birmingham, AL, United States; ^2^Department of Neurology, University of Alabama at Birmingham, Birmingham, AL, United States; ^3^School of Medicine, University of Alabama at Birmingham, Birmingham, AL, United States; ^4^Department of Psychology and Neuroscience, University of California, Berkeley, Berkeley, CA, United States; ^5^Department of Neurology and Neurosurgery, University of California, San Francisco, San Francisco, CA, United States

**Keywords:** deep brain stimulation, intermittent theta-burst stimulation, subthalamic nucleus, globus pallidus interna, Parkinson’s disease, cognition

## Abstract

**Introduction:**

Cognitive symptoms from Parkinson’s disease cause severe disability and significantly limit quality of life. Little is known about mechanisms of cognitive impairment in PD, although aberrant oscillatory activity in basal ganglia-thalamo-prefrontal cortical circuits likely plays an important role. While continuous high-frequency deep brain stimulation (DBS) improves motor symptoms, it is generally ineffective for cognitive symptoms. Although we lack robust treatment options for these symptoms, recent studies with transcranial magnetic stimulation (TMS), applying intermittent theta-burst stimulation (iTBS) to dorsolateral prefrontal cortex (DLPFC), suggest beneficial effects for certain aspects of cognition, such as memory or inhibitory control. While TMS is non-invasive, its results are transient and require repeated application. Subcortical DBS targets have strong reciprocal connections with prefrontal cortex, such that iTBS through the permanently implanted lead might represent a more durable solution. Here we demonstrate safety and feasibility for delivering iTBS from the DBS electrode and explore changes in DLPFC electrophysiology.

**Methods:**

We enrolled seven participants with medically refractory Parkinson’s disease who underwent DBS surgery targeting either the subthalamic nucleus (STN) or globus pallidus interna (GPi). We temporarily placed an electrocorticography strip over DLPFC through the DBS burr hole. After placement of the DBS electrode into either GPi (*n* = 3) or STN (*n* = 4), awake subjects rested quietly during iTBS (three 50-Hz pulses delivered at 5 Hz for 2 s, followed by 8 s of rest). We contrasted power spectra in DLPFC local field potentials during iTBS versus at rest, as well as between iTBS and conventional high-frequency stimulation (HFS).

**Results:**

Dominant frequencies in DLPFC at rest varied among subjects and along the subdural strip electrode, though they were generally localized in theta (3–8 Hz) and/or beta (10–30 Hz) ranges. Both iTBS and HFS were well-tolerated and imperceptible. iTBS increased theta-frequency activity more than HFS. Further, GPi stimulation resulted in significantly greater theta-power versus STN stimulation in our sample.

**Conclusion:**

Acute subcortical iTBS from the DBS electrode was safe and well-tolerated. This novel stimulation pattern delivered from the GPi may increase theta-frequency power in ipsilateral DLPFC. Future studies will confirm these changes in DLPFC activity during iTBS and evaluate whether they are associated with improvements in cognitive or behavioral symptoms from PD.

## Introduction

Deep brain stimulation (DBS) is an established therapy for Parkinson’s disease (PD) and other movement disorders (Deuschl et al., 2006; Starr et al., 2006; Baizabal-Carvallo et al., 2014). However, standard DBS is not generally considered effective for the cognitive impairments associated with PD (Cernera et al., 2019), which can be a source of overwhelming disability (Duncan et al., 2014). A small number of studies have suggested that novel DBS paradigms may address this issue in PD and other diseases. For example, theta-range (5–8 Hertz [Hz]) DBS appears to improve measures of inhibitory control and interval timing accuracy (Kelley et al., 2018; Scangos et al., 2018). DBS in other neural targets, such as the fornix, is also under investigation for the cognitive symptoms of Alzheimer’s disease (Lozano et al., 2016, 2019). Support for the possibility of DBS affecting cortical cognitive networks is in part derived from studies showing DBS effects on primary motor areas. Clinically effective high-frequency DBS at subcortical targets for movement disorders [subthalamic nucleus (STN), globus pallidus interna (GPi)] results in beta-oscillation desynchronization and reduced phase-amplitude coupling (Asanuma et al., 2006; De Hemptinne et al., 2015). However, much less is known about possible interactions with prefrontal cortical areas using novel parameters. If these interactions occur, it would serve as a foundation for optimization of next-generation devices aimed at improving not only motor symptoms, but also cognitive effects of the disease as well.

Previous studies investigating the potential role of stimulation for cognition have primarily used theta-frequency pulses, which underlies many cognitive processes, especially in prefrontal cortex (Canolty et al., 2006; Cavanagh and Frank, 2014; Helfrich and Knight, 2016). Among the various prefrontal regions involved, the dorsolateral prefrontal cortex (DLPFC, Brodmann areas 9 and 46) is of special interest in PD as it is active during reward learning, set-shifting, action selection (Ridderinkhof et al., 2004), and inhibitory control (MacDonald et al., 2000; Harrison et al., 2005; Oldrati et al., 2016), which PD patients have particular difficulty with (Manza et al., 2017). The DLPFC has direct connections to the STN (Haynes and Haber, 2013) and GPi (Middleton and Strick, 2002), as well as widespread connections to the caudate nucleus and to the orbitofrontal, cingulate, pre-motor, and pre-supplementary motor cortices (Ridderinkhof et al., 2004). In PD patients, functional magnetic resonance imaging (fMRI) studies reveal reduced DLPFC activity during inhibitory control tasks, with increased activity after administration of anti-Parkinsonian medications correlating to improved inhibitory control task performance (Trujillo et al., 2019). Furthermore, EEG studies show that theta-frequency activity is decreased in PD patients performing these tasks (Singh et al., 2018).

It follows that increasing theta-power in impaired individuals may improve cognitive function. Recent studies from the transcranial non-invasive stimulation literature are providing some insight into how this might be achieved. For example, theta-frequency transcranial alternating-current stimulation (tACS) improved working memory in healthy older adults (Reinhart and Nguyen, 2019). An emerging therapy that shows promise for improving cognition that is now Food and Drug Administration (FDA) approved for depression (Blumberger et al., 2018) is intermittent theta-burst stimulation (iTBS), delivered via transcranial magnetic stimulation (TMS) to the prefrontal cortex (Hoy et al., 2016; Lowe et al., 2018). This form of therapy is thought to mimic natural brain activity, and in addition to enhancing memory in healthy adults (Hoy et al., 2016; Reinhart and Nguyen, 2019), it may also have effects on cognitive function in PD (Benninger et al., 2011; Dinkelbach et al., 2017; Trung et al., 2019). However, the effects of TMS are transient, requiring frequent re-application. Delivery of iTBS through a DBS lead implanted in subcortical sites which are already approved for therapy could represent a more durable solution. To this end, it is important to determine whether DBS at these sites can modulate DLPFC activity, whether through iTBS or standard high-frequency stimulation (HFS).

Here, we implant unilateral DBS electrodes into GPi or STN in PD patients, deliver both conventional high-frequency stimulation (>100 Hz) and iTBS, and record intracranial local field potentials (LFPs) from DLPFC with a subdural strip electrode. We report on the safety and feasibility of this approach and describe changes in theta and alpha/beta power in DLPFC between stimulation conditions, from both GPi and STN.

## Materials and Methods

### Patient Selection

Participants were diagnosed with PD by a movement disorders neurologist and deemed candidates for DBS surgery after consensus review at a multi-disciplinary conference of neurologists, neurosurgeons, neuropsychologists, and nurse practitioners. Stimulation target (STN or GPi) was chosen based on clinical features. All research procedures were approved by the University of Alabama at Birmingham Institutional Review Board with written informed consent.

### Surgical Procedure

All participants underwent three Tesla MR imaging (Magnetom PRISMA, Siemens Healthcare GmbH, Erlangen, Germany) with the exception of Subject 4 who instead had high resolution CT imaging because of a contraindication to MRI (metal implant). DBS surgery was performed in the awake, off-medication state, at least 12 h following medication administration. A stereotactic headframe was placed (Cosman-Roberts-Wells, Integra LifeSciences, Plainsboro, NJ, United States), and an intraoperative 3D fluoroscopic image was obtained (O-arm 2, Medtronic, Minneapolis, MN, United States) and merged to pre-operative MRI. The prescribed target was identified according to standard techniques. To localize the DLPFC, we identified the mid-portion of the middle frontal gyrus along its longitudinal axis anterior to the pre-motor area (Trujillo et al., 2019), and designated this point as the midpoint for the subdural electrode. A radiopaque marker (18G needle) was placed at this point using the stereotactic headframe for localization.

After creating the burr hole and opening the outer dural layer but prior to DBS lead placement, we placed a 6-contact subdural strip electrode (Ad-tech Medical, Oak Creek, WI, United States) over the cortical surface, guided toward the scalp marker under X-ray fluoroscopic guidance. We then continued with the DBS procedure as routinely performed, beginning with microelectrode recordings. After defining the optimal location for the DBS, we performed 3D fluoroscopy to confirm our location. This image was merged intra-operatively to the pre-operative planning MRI to confirm subdural strip placement. We placed the DBS lead at its final position, then performed clinical testing for side effects and efficacy. Following this, the research paradigm began. After completion of the research testing (approximately 10–15 min), we removed the subdural strip electrode and proceeded with securing of the DBS lead and closure.

### DLPFC Recordings

We recorded local field potentials from the subdural strip electrode over DLPFC with an actiCHamp active channel amplifier (BrainVision, Morrisville, NC, United States), sampling at 25 kHz with an analog 7.5 kHz low-pass filter and no further digital filters. We placed ground and reference EEG electrodes on the on the forehead and contralateral mastoid, respectively, and recorded muscle activity from the contralateral hand and forearm with bipolar EMG pad electrodes to screen for unwanted, incidental movements during recordings. Recordings were obtained with subjects awake, quiet, and at rest, first without stimulation, then with HFS and iTBS.

### Subcortical Stimulation

**FIGURE 1:**
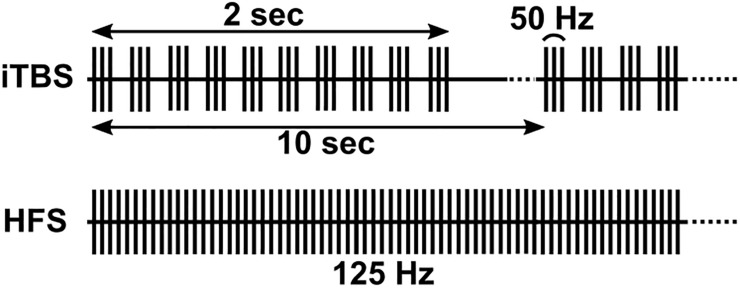
Illustration of iTBS and HFS paradigms. iTBS consists of ten bursts of three pulses at 50 Hz (lasting 2 s), repeated at 10 s intervals. HFS consisted of a constant 125 Hz stimulation.

Biphasic square waves were delivered through the DBS lead via an external pulse generator (STG4008, MultiChannel Systems, Reutlingen, Germany) following routine clinical macrostimulation, typically with a bipolar configuration of contacts 3 and 0, and amplitude and pulse width that conferred robust clinical benefit during behavioral testing with DBS at 160 Hz. To mark stimulus times, the STG4008 delivered a TTL pulse for each stimulus to the recording amplifier. During HFS, stimuli were delivered continuously at 125 Hz for 2 min (Figure 1, bottom). We delivered iTBS using standard parameters from the TMS literature (10 bursts of 3 stimulus pulses at 50 Hz, each burst separated by 200 ms [5 Hz], repeated over 2 s followed by an 8 s period of rest. This pattern was then repeated over 2 min (Figure 1, top). Pulse widths were based on the TMS literature for Subject 2 (300 μs) and were decreased to standard DBS pulse widths for all subsequent participants (60 μs). In one participant (Subject 4, bilateral hemispheres) we administered 4 Hz continuous stimulation for comparison to iTBS, in lieu of HFS (Kelley et al., 2018).

### Post-operative Electrode Localization

We visualized DLPFC by first extracting a 3D model of the cortical surface from pre-operative MRI with FreeSurfer (Fischl, 2012). DLPFC was then identified as the combination of the rostral and caudal middle frontal regions, as labeled by FreeSurfer based on the Desikan-Killiany atlas. To localize the subdural strip electrodes, the intra-operative CT, pre-operative MRI, and 3D cortical model were imported into 3D Slicer (Fedorov et al., 2012). CT images were co-registered with the MRI and cortical model using an affine transform in the “General Registration (BRAINS)” module. Virtual fiducial markers then were manually placed in the center of the artifact of each strip contact. All contacts could be easily identified in each case and reconstructed, with the exception of Subject 4 who did not have an MRI available for reconstruction.

### Signal Processing and Local Field Potential Analysis

All signal processing and statistical analyses were performed in MATLAB R2018b (MathWorks, Natick, MA, United States). Signals recorded from the subdural strip were downsampled to 400 Hz after applying a second order 1.5–75 Hz Butterworth filter. Individual channels (referenced to the contralateral mastoid) displaying high noise and/or overwhelming electrical artifacts were excluded from further analysis. The remaining channels were then re-referenced to a common average montage.

Power spectra were estimated as a global wavelet spectrum from each channel with and without stimulation, by averaging the continuous wavelet transform (CWT) across time during HFS and iTBS. For all analyses utilizing the CWT, the following parameters were used: complex morse wavelets, time-bandwidth product of 120, and 20 voices per octave.

To measure changes in spectral power during stimulation while allowing for inter-subject differences in frequency distributions and contact locations, ECoG contacts were first grouped into three general locations: anterior (contacts 1 and 2), middle (contacts 3 and 4), or posterior (contacts 5 and 6). The power spectra of the two component contacts in each group were then averaged together, and peaks in either the theta or alpha/beta frequency ranges were identified in the stimulation period using the “findpeaks” function in MATLAB. The width of the maximum peak was estimated as the point of half-prominence on either side, as determined by the same function. The mean power in this band was then computed by averaging the continuous wavelet transform across the band and across time. Finally, this mean power was converted to a *Z*-score by subtracting the mean of the same band during the corresponding no-stimulation baseline period and dividing by the standard deviation. Thus, we were able to quantify the impact of stimulation across subjects, DBS targets, and contact location groups.

## Results

### Patient Demographics and Stimulation Parameters

Patient demographics and stimulation parameters are summarized in Table 1. Seven subjects underwent awake unilateral DBS surgery for Parkinson’s disease, one of whom underwent contralateral DBS implantation in a subsequent surgery, for a total of eight DBS electrodes placed either in STN (*n* = 5) or GPi (*n* = 3). Resting DLPFC LFPs were recorded from all subjects (*n* = 8 hemispheres), and we delivered HFS from the DBS electrode in four participants (*n* = 4 hemispheres), 4-Hz continuous stimulation in 1 participant (*n* = 1 hemisphere) and iTBS in six participants (*n* = 7 hemispheres). Mean age at surgery was 69.4 years (S.D. 7.3, range 55–76 years). Mean duration of disease was 7.7 years (S.D. 3.7, range 5–15 years), with 71.4% (5/7) right-handed individuals and 1 ambidextrous subject. The right hemisphere was targeted in 62.5% of recordings. In all patients, a 6-contact subdural strip was placed over DLPFC without adverse effects. Stimulation was delivered as previously described, with HFS delivered at 125 Hz with bipolar contact pairs, at 60 μs pulse widths, and ranging from 2.0 to 6.0 milliamperes (mA), as summarized in Table 1. We delivered iTBS from the same bipolar contact pair, and at the same current and pulse width, that was used for HFS. In participant 2, in whom HFS was not applied, we applied the current at which clinical benefit was seen. The pulse width was the same as used in TMS studies of iTBS, though for subsequent participants we used a narrower pulse width to reduce charge density.

**TABLE 1:** Patient demographics and stimulation parameters.

Subject	Age at	Disease	Target	Hemisphere	Stimulation	Stimulation	Stimulation pulse
No.	surgery* (yrs)	Duration (yrs)			type	amplitude (mA)	width (us)
1	70–75	5	STN	Right	None		
2	70–75	6	GPi	Right	iTBS^†^	4.5	300
3	70–75	5	GPi	Left	iTBS, HFS^‡^	2.0	60
4^§^	75–80	9	STN	Left/Right	iTBS, 4-Hz	4.6/4.0	60
5	75–80	15	STN	Right	iTBS, HFS	5.0	60
6	55–60	5	STN	Left	iTBS, HFS	3.2	60
7	65–70	9	GPi	Right	iTBS, HFS	6.0	60

*Ages presented as ranges to avoid identifiable participant data. ^†^iTBS frequency parameters: Three 50 Hz pulses at 5 Hz for 2 s, followed by 8 s of rest; repeated over 2 min. ^‡^HFS frequency parameters: Continuous 125 Hz. ^§^Participant 4 initially had DBS implanted in the left STN and subsequently had the right STN implanted 2.5 months later. DLPFC recording and iTBS were performed in both surgeries. STN, subthalamic nucleus; GPi, Globus pallidus interna; iTBS, intermittent theta-burst stimulation; HFS, high-frequency stimulation; mA, milliamperes; μs, microseconds.

### Resting Peak Frequencies in DLPFC Varies Across Subjects and Across Contacts

**FIGURE 2:**
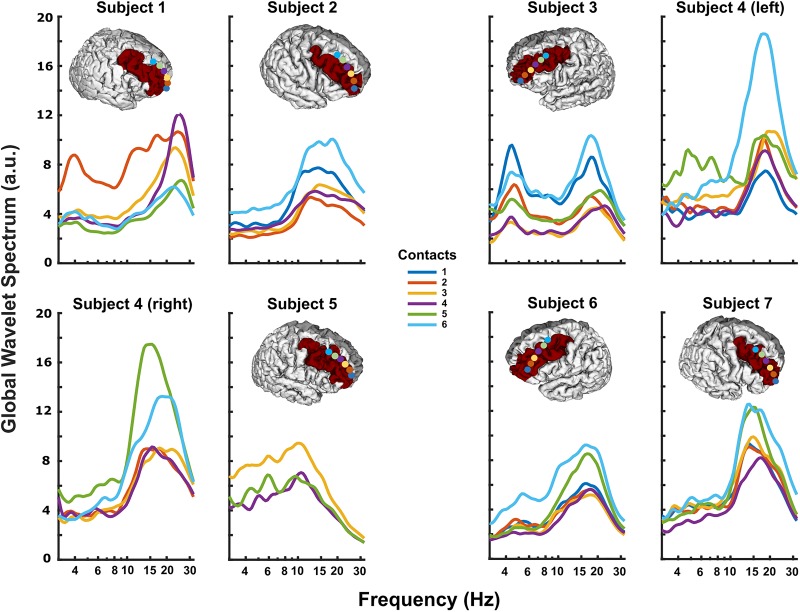
Resting DLPFC local field potentials recorded from each subject. The 3D reconstruction of each subject’s cortical surface (except Subject 4), with localized subdural strip contacts (circles colored according to contact number) and DLPFC region colored red. All subjects displayed prominent peaks in theta (3–8 Hz) and/or alpha/beta (10–30 Hz) ranges. In some subjects, particularly Subject 1, theta and alpha/beta activity had clearly different distributions along the strip, possibly indicating separate neural sources.

DLPFC power spectra at rest typically displayed prominent peaks in theta (3–8 Hz) (Subjects 1, 3, 4-left, 5, and 6; Figure 2) and/or alpha/beta range (10–30 Hz) (all subjects; Figure 2). Spectral power varied systematically across the subdural strip, with more prominent theta at the rostral and/or caudal extremes versus the middle contacts (e.g., subjects 1 and 3 in Figure 2). Alpha/beta peaks were more variably distributed across the strip, but tended to have highest power in the more caudal contacts, nearest pre-motor cortex (e.g., subjects 4-L, 4-R, and 7 in Figure 2). Theta and alpha/beta peaks appeared to arise from different contacts, although in two subjects the maximal peaks for these two frequency bands were in the same contacts.

### iTBS From the GPi Modulates DLPFC Theta-Frequency LFP

**FIGURE 3:**
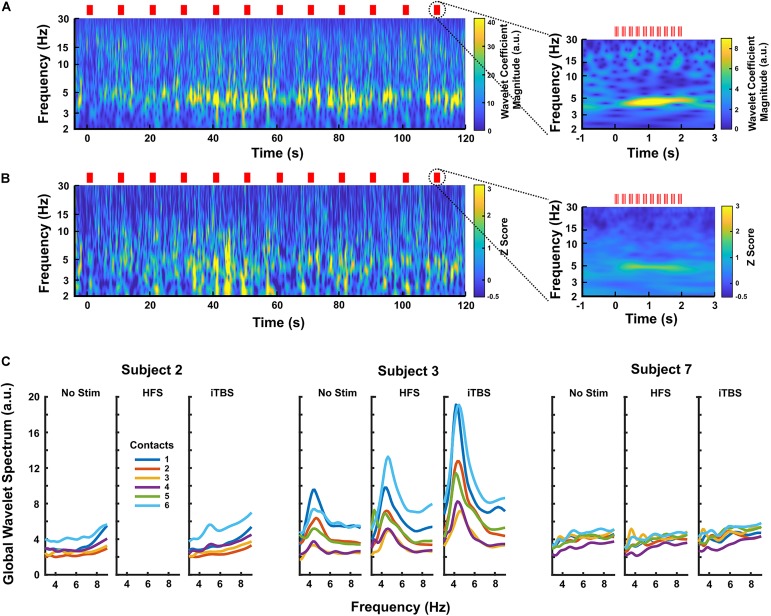
**(A)** Continuous wavelet transform scalogram showing an example of the effects of iTBS on DLPFC LFPs in Subject 3 (contact 6, the most caudal contact on the subdural strip). Red lines mark stimulus times, and the start of stimulation is aligned at zero. Increased theta activity is prominent during iTBS, increasing after a delay of ∼30 s. The inset shows the LFP activity averaged across each 2 s block of theta bursts (*n* = 20 blocks). Here, the theta increase is clearly time-locked to the stimulation, appearing to build up over a period of ∼0.5 s. **(B)** Scalogram showing the effects of iTBS on DLPFC LFPs averaged over all three GPi subjects. Each subject’s scalogram was converted to a *Z*-score based on that subject’s baseline recordings before being averaged. The most caudal contact, contact 6, was used for each subject. Again, the inset shows the activity averaged over each 2 s block of theta bursts (*n* = 59 blocks), demonstrating that time-locking is preserved across subjects. **(C)** Average power spectra for all contacts in each GPi subject during no-stimulation, HFS, and iTBS periods, showing that iTBS increases theta activity more than does HFS on the same contacts. Subject 3 had the largest response to stimulation, but Subject 2 displayed a clear rise in 5 Hz activity. Subject 7 had minimal response to any stimulation condition.

When iTBS was delivered subcortically, temporally related changes were seen in the DLPFC (Figure 3). This finding was most pronounced in Subjects 2 and 3, with Subject 7 having little clear change. These increases in theta band power were delayed by approximately 30 s relative to the start of iTBS (Figures 3A,B). When the LFPs recorded during each set of 10 iTBS bursts within a subject were averaged together to create a mean event-related wavelet spectrogram, the increase in theta power was delayed by approximately 0.5 s and time-locked to the burst onset (Figures 3A,B, insets). Notably, we did not see significant activity evoked by single pulses at either target, and high-frequency stimulation did not elicit these changes (Supplementary Figure S1).

**FIGURE 4:**
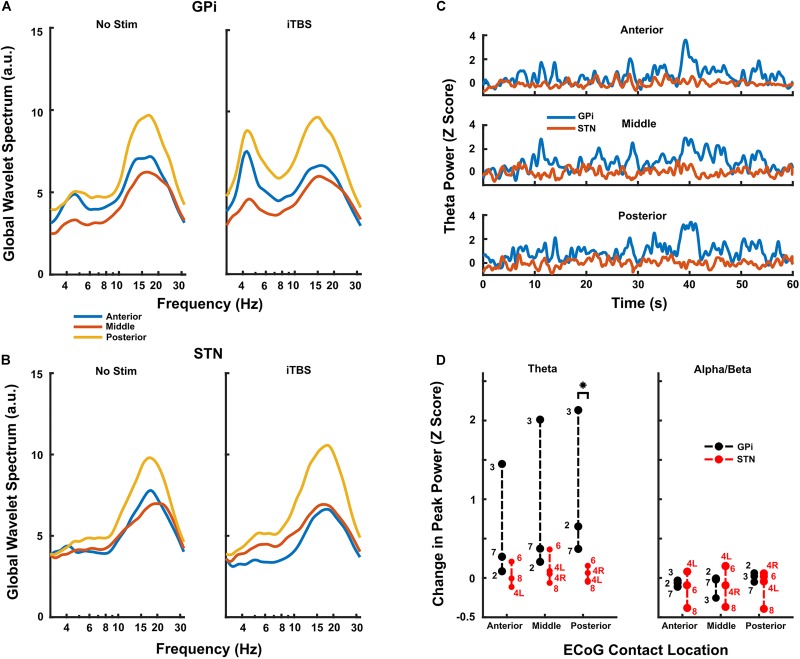
**(A,B)** Resting and iTBS power spectra for each contact location group, averaged across all GPi **(A)** and STN **(B)** subjects. There was a clear trend toward higher theta facilitation caudally on the strip in the GPi subjects and there was minimal evidence of facilitation in the STN group. **(C)** Time series of theta activity in each contact location group during iTBS, averaged across GPi (blue) and STN (red) subjects. Each subject’s activity was first converted to a *Z*-score based on the baseline theta activity prior to being averaged. Stimulation starts at time 0. Traces show high variability typical of neural data, but clearly show the differences in changes induced by iTBS delivered in GPi versus STN. **(D)** Group data for all subjects (*n* = 3 GPi; *n* = 4 STN) undergoing iTBS. In the posterior contact group, there was a significant difference in facilitation of theta power (compared to baseline) when iTBS was delivered in GPi versus STN (*p* = 0.0286, Wilcoxon rank-sum test). Conversely, the theta facilitation did not reach significance in any other contact group. There was no statistical difference in facilitation of beta activity in any contact group, and there was clearly less change overall compared to theta activity. Subject numbers appear next to each point.

When comparing DLPFC changes by target, iTBS increased DLPFC theta-frequency activity to a greater extent during GPi (*n* = 3) versus STN (*n* = 4) stimulation when normalized versus rest (Figure 4, *p* = 0.0286 at the posterior contact group, Wilcoxon rank-sum test). This difference was most pronounced in the contacts over posterior DLPFC. Less pronounced changes in theta-power occurred in contacts over anterior and middle DLPFC, though theta-power still generally increased to a greater extent with GPi versus STN stimulation. Although a small effect, STN stimulation may have even decreased cortical theta power slightly in 1-2 subjects (Figure 4D). No clear differences in alpha/beta-power changes were observed between targets (Figure 4, *p* = 0.314 at the posterior contact group, Wilcoxon rank-sum test), though power decreased in several subjects and in several contact locations. Since only two GPi subjects underwent both HFS and iTBS, we did not similarly contrast LFPs during HFS between targets. However, in both cases, HFS increased DLPFC theta-frequency power less than did iTBS (Supplementary Figure S2).

## Discussion

New DBS technologies for movement disorders are developing at a rapid pace, with directional leads capable of current steering (Pollo et al., 2014; Dembek et al., 2017) and recording and sensing devices under investigation for closed-loop control (Rosin et al., 2011; Priori et al., 2013; Swann et al., 2018). Given this pace of device development, it may be possible that future iterations can incorporate multiple stimulation patterns addressing multiple symptoms of these diseases. In PD, non-motor cognitive symptoms are highly prevalent and disabling (Hely et al., 2008), with pronounced deficits in attention, memory, visuospatial processing, and response inhibition (Williams-Gray et al., 2009; Antonini et al., 2012; Svenningsson et al., 2012; Duncan et al., 2014; Manza et al., 2017). Medical treatments fail to improve cognitive symptoms in many patients (Svenningsson et al., 2012), and cognitive outcomes following conventional high-frequency DBS are mixed (Okun et al., 2009; Combs et al., 2015; Wang et al., 2016), with a recent meta-analysis finding that STN-DBS patients experienced decrements in multiple cognitive domains compared to medically-treated controls (Cernera et al., 2019). Ideally, next-generation therapies would address both motor and cognitive aspects of the disease, but will likely require alternative patterns of stimulation.

Efficient cognitive processing likely involves coordinated signaling across multiple areas in distributed networks (Medaglia et al., 2015; Helfrich and Knight, 2016), with the prefrontal cortex acting as a major hub for many of these processes (Aron, 2007; Voytek and Knight, 2015). In particular, the DLFPC is consistently activated in cognitive tasks, including set-shifting, action selection, reward learning (Ridderinkhof et al., 2004) and tasks of inhibitory control (MacDonald et al., 2000; Harrison et al., 2005; Oldrati et al., 2016). As PD patients have deficits in several of these cognitive domains (Obeso et al., 2011; Manza et al., 2017), it is hypothesized that DLPFC function is correlated to impairment in these individuals. This is supported by both fMRI and EEG studies that reveal hypoactivity in the DLPFC of these patients (Schmiedt-Fehr et al., 2007; Singh et al., 2018; Trujillo et al., 2019).

Mechanistically, aberrant function of the DLPFC may be due to abnormal theta-frequency activity, which is thought to underlie intact cognitive processes (Cavanagh and Frank, 2014). Notably, patients with PD have reduced theta-rhythms during cognitive tasks, particularly those involving inhibitory control (Schmiedt-Fehr et al., 2007; Singh et al., 2018). Therefore, restoring “normal” theta activity may result in improved task performance. To this end, several DBS studies have investigated subcortical delivery of continuous theta-frequency stimulation. For example, 4-Hz and 5-Hz continuous DBS is associated with improvement in interval timing (Kelley et al., 2018) and Stroop tasks (Scangos et al., 2018), respectively, and fornix stimulation is currently under investigation for memory improvement in Alzheimer’s disease (Lozano et al., 2016). Non-invasive techniques are also becoming more widely studied. In transcranial magnetic stimulation (TMS), in which magnetic pulses are delivered through the scalp to interact with neural firing, several modes of theta-stimulation have been tried, including continuous and intermittent bursting patterns (Viejo-Sobera et al., 2017; Lowe et al., 2018). Theta-burst stimulation is thought to mimic naturally occurring brain rhythms (Huang and Rothwell, 2004) and in intermittent theta-burst stimulation, three pulses are delivered at 50-Hz every 200 ms for 2 s, followed by 8 s of rest (Huang et al., 2005). Initial effects were seen when delivered over motor cortex (Huang and Rothwell, 2004; Huang et al., 2005), and since then, it has been increasingly used to modulate cognitive networks (Hoy et al., 2016; Chung et al., 2018; Ji et al., 2019; Trung et al., 2019). However, a recent study of iTBS in PD showed failure to improve frontal executive function and memory when delivered via TMS, which suggests a single session of therapy is not sufficient (Hill et al., 2020). Multiple sessions may provide benefit (Trung et al., 2019), but frequent re-application may not be logistically feasible for patients (Dinkelbach et al., 2017). In addition, the field of spread of the TMS pulse is variable due to tissue inhomogeneity, reducing the precision and predictability of this technique (Opitz et al., 2011). For these reasons, further studies of delivering iTBS patterns using deep brain stimulation are warranted.

Theta-burst patterns have previously been delivered via deep brain stimulation, primarily in the context of stimulation for memory improvement (Suthana et al., 2012; Titiz et al., 2017). Suthana et al. performed double-blinded theta-burst stimulation in the entorhinal cortex and hippocampus of 7 epilepsy patients and found improvement in a spatial learning task (Suthana et al., 2012). Similarly, in a double-blinded study of four patients, Miller et al. delivered theta-burst stimulation to the fornix of the hippocampus via depth electrodes, with overall improved performance (Miller et al., 2015), replicating prior results in animal models (Sweet et al., 2014).

In order to test the feasibility of subcortical iTBS, we delivered this pattern of stimulation in 7 PD patients undergoing routine DBS surgery. We show for the first time in humans that iTBS can be safely delivered and further show that GPi, but not STN, stimulation appears to modulate DLPFC theta activity, though responses across subjects and across anatomic areas were variable. Our results also indicate that high-frequency stimulation itself does not clearly modulate theta-power, and neither iTBS nor HFS had a substantial effect on other frequency bands. Due to the increase of theta power seen in the DLPFC of some PD patients, the implication is that subcortical iTBS may be useful for enhancing oscillatory activity and potentially correlate with cognitive improvement in impaired individuals.

Overall, this study provides evidence for the safety and feasibility of this approach, and provides some indication that iTBS may prove useful for modulating prefrontal cognitive networks. Further investigation is required to determine if increased theta-power correlates to behavioral changes in cognitive domains. If supported, this could serve as a foundation for developing next-generation DBS technologies for addressing non-motor cognitive and behavioral symptoms of Parkinson’s disease and other disorders.

### Limitations

Due to the small sample size, statistical tests were limited. However, placement of the strip electrode over DLPFC and iTBS were well-tolerated in all subjects, and our results reached statistical significance with regard to changes in theta-power during iTBS by stimulation target. A larger sample is required to form conclusion about connectivity between DLPFC and subcortical networks, especially given the variation in responses to stimulation within and across GPi implants. Although we did not correlate theta-activity with behavioral measures, these types of studies represent an important next step for this research. Finally, artifacts are always of consideration when interpreting recorded brain activity. It may be argued that the observed increased theta-power is an artifact of volume conduction from subcortical stimulation. However, we believe this is not the case since high-frequency stimulation did not result in analogous artifacts. Additionally, the electrical artifacts from the stimulation were limited to frequencies >100 Hz and would not have impacted theta-frequency activity.

## Conclusion

Here, we show that iTBS, a type of patterned stimulation that is increasingly being investigated for cognitive and behavioral therapies via TMS, can be safely delivered from subcortical structures routinely targeted for DBS therapy in Parkinson’s disease. As far as we are aware, this is the first demonstration of subcortical iTBS in humans. In our sample, we also show that iTBS from the GPi, but not the STN, appears to drive theta-frequency activity in the DLPFC. This is of interest since theta oscillatory activity may play a role in aberrant cognitive processing in PD. Further studies are required to confirm this result and determine if increasing theta activity in the DLPFC correlates with behavioral changes.

## Data Availability Statement

The datasets generated for this study are available on request to the corresponding author.

## Ethics Statement

The studies involving human participants were reviewed and approved by the University of Alabama, Birmingham Institutional Review Board. The patients/participants provided their written informed consent to participate in this study.

## Author Contributions

JB, ZI, RK, BG, and HW contributed to conception and design of the study. JB, ZI, MR, CG, and HW contributed to data collection. SB performed electrode localizations. NB and ZI wrote the first draft of the manuscript. AK and GE-S wrote sections of the manuscript. All authors contributed to the manuscript revision, read, and approved the submitted version.

## Conflict of Interest

The authors declare that the research was conducted in the absence of any commercial or financial relationships that could be construed as a potential conflict of interest.

## Supplementary Material

FIGURE S1 Power spectra for each stimulation condition performed in each subject. Contact 1 is the most anterior and contact 6 is the most posterior in all subjects.

FIGURE S2 Comparison of iTBS versus HFS in all GPi subjects. Only Subjects 3 and 7 underwent both protocols.
