# Supplementary figures and images for: Subcortical Intermittent Theta-Burst Stimulation (iTBS) Increases Theta-Power in Dorsolateral Prefrontal Cortex (DLPFC)
Source: Front Neurosci. 2020 Jan 31;14:41. doi: 10.3389/fnins.2020.00041 (PMC7006239; doi:10.3389/fnins.2020.00041)

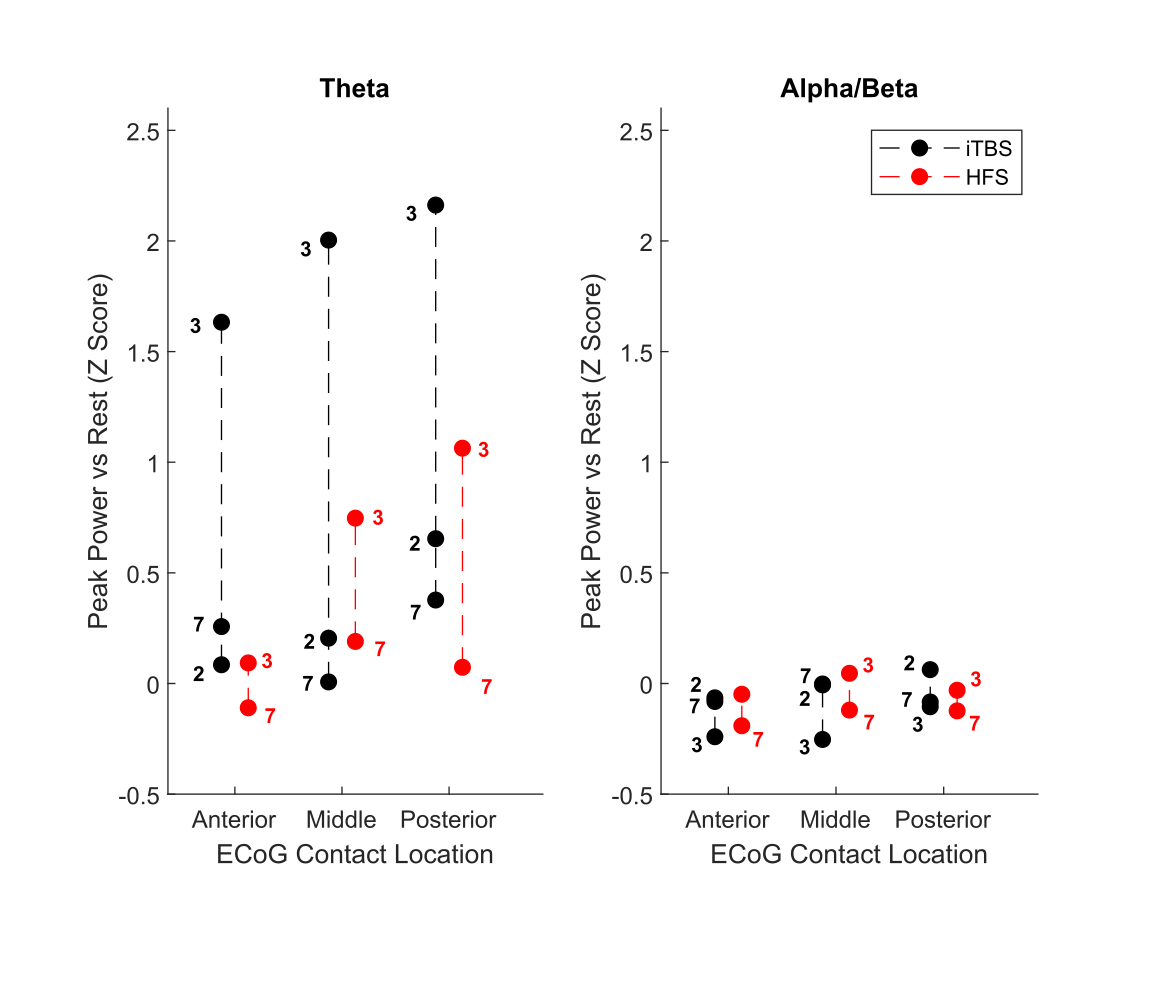

Supplement: FIGURE S1 — Power spectra for each stimulation condition performed in each subject. Contact 1 is the most anterior and contact 6 is the most posterior in all subjects. [file Image_1.tif]

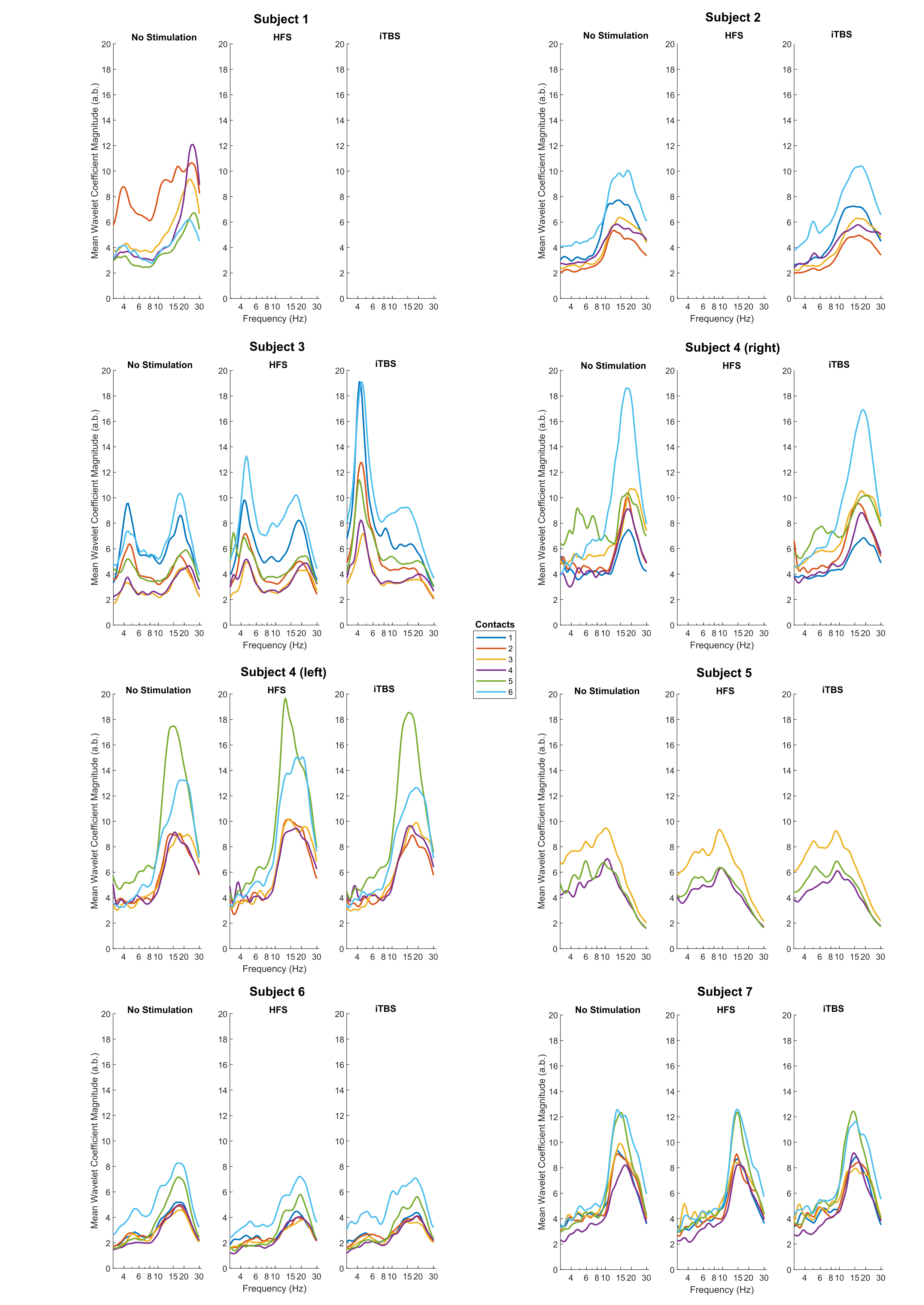

Supplement: FIGURE S2 — Comparison of iTBS versus HFS in all GPi subjects. Only Subjects 3 and 7 underwent both protocols. [file Image_2.tif]
